# Drug allergy

**DOI:** 10.1186/1710-1492-7-S1-S10

**Published:** 2011-11-10

**Authors:** Richard Warrington, Fanny Silviu-Dan

**Affiliations:** 1 University of Manitoba, Winnipeg, Manitoba, Canada; 2 McGill University, Montreal, Quebec, Canada

## Abstract

Drug allergy encompasses a spectrum of immunologically-mediated hypersensitivity reactions with varying mechanisms and clinical presentations. This type of adverse drug reaction (ADR) not only affects patient quality of life, but may also lead to delayed treatment, unnecessary investigations, and even mortality. Given the myriad of symptoms associated with the condition, diagnosis is often challenging. Therefore, referral to an allergist experienced in the identification, diagnosis and management of drug allergy is recommended if a drug-induced allergic reaction is suspected. Diagnosis relies on a careful history and physical examination. In some instances, skin testing, graded challenges and induction of drug tolerance procedures may be required.

The most effective strategy for the management of drug allergy is avoidance or discontinuation of the offending drug. When available, alternative medications with unrelated chemical structures should be substituted. Cross-reactivity among drugs should be taken into consideration when choosing alternative agents. Additional therapy for drug hypersensitivity reactions is largely supportive and may include topical corticosteroids, oral antihistamines and, in severe cases, systemic corticosteroids. In the event of anaphylaxis, the treatment of choice is injectable epinephrine. If a particular drug to which the patient is allergic is indicated and there is no suitable alternative, induction of drug tolerance procedures may be considered to induce temporary tolerance to the drug.

This article provides a backgrounder on drug allergy and strategies for the diagnosis and management of some of the most common drug-induced allergic reactions, such allergies to penicillin, sulfonamides, cephalosporins, radiocontrast media, local anesthetics, general anesthetics, acetylsalicylic acid (ASA) and non-steroidal anti-inflammatory drugs.

## Introduction

Adverse drug reactions (ADRs) are defined as any harmful or unintended reaction to a drug that occurs at doses used for prevention, diagnosis, or treatment [1]. ADRs are common in everyday clinical practice, affecting between 15-25% of patients; serious reactions occur in 7-13% of patients [2,3].

ADRs are classified as either predictable reactions that may occur in anyone (type A) or unpredictable reactions that occur in only susceptible individuals (type B) (see Table 1). Predictable reactions are the most common type of ADR and are usually dose dependent and related to the known pharmacologic actions of the drug (e.g., side effects, overdose, drug interactions). Unpredictable reactions occur in approximately 20-25% of patients who experience ADRs; these reactions are generally unrelated to the pharmacologic actions of the drug [1,4,5].

**Table 1:** Classification of adverse drug reactions [1,4,5]

Type A: Predictable	Type B: Unpredictable
• Drug overdose • Secondary drug effects • Side effects • Drug interactions	*• Drug allergy:* an immunologically mediated ADR *• Pseudoallergic* (*non-allergic*)*:* a reaction with the same clinical manifestations as an allergic reaction, but that lacks immunological specificity *• Drug intolerance:* an undesirable pharmacologic effect that occurs at low and sometimes sub-therapeutic doses of the drug that are not caused by underlying abnormalities of metabolism or drug excretion *• Drug idiosyncrasy:* an abnormal/unexpected effect, usually caused by underlying abnormalities of metabolism, excretion, or bioavailability

ADR: adverse drug reaction

Drug allergy is one type of unpredictable ADR that encompasses a spectrum of immunologically-mediated hypersensitivity reactions with varying mechanisms and clinical presentations [1]. It accounts for approximately 5-10% of all ADRs [6]. Pseudoallergic reactions (also known as non-allergic or non-immune-mediated reactions) represent another type of unpredictable ADR. These reactions are often indistinguishable from true immunologically mediated allergic reactions, but they lack immunological specificity.

Drug allergy not only affects patient quality of life, but may also lead to delayed treatment, use of suboptimal alternate medications, unnecessary investigations and even death. Furthermore, the identification of drug allergy is challenging given the myriad of symptoms and clinical presentations associated with the condition. Therefore, if a drug-induced allergic disorder is suspected, consultation with an allergist experienced in the identification, diagnosis and management of drug allergy is recommended. This article will provide an overview of the mechanisms and risk factors for drug allergy, as well as strategies for the diagnosis and appropriate management of some of the most common drug-induced allergic disorders.

## Mechanisms

Immune-mediated allergic reactions to drugs are classified according to Gell and Coombs’ classification system, which describes the predominant immune mechanisms involved in these reactions. This classification system includes: immediate-type reactions mediated by immunoglobulin E (IgE) antibodies (type I), cytotoxic reactions mediated by immunoglobulin G (IgG) or immunoglobulin M (IgM) antibodies (type II), immune-complex reactions (type III), and delayed-type hypersensitivity reactions mediated by cellular immune mechanisms, such as the recruitment and activation of T cells (type IV) [7-9]. The mechanisms, clinical manifestations, and timing of these immune reactions are summarized in Table 2.

**Table 2:** Classification of allergic drug reactions: mechanisms, clinical manifestations, and timing of reactions. [7-9]

Immune reaction	Mechanism	Clinical manifestations	Timing of reaction
Type I (IgE-mediated)	Drug-IgE complex binding to mast cells with release of histamine, inflammatory mediators	Anaphylaxis*, urticaria*, angioedema*, bronchospasm*	Minutes to hours after drug exposure

Type II (cytotoxic)	Specific IgG or IgM antibodies directed at drug-hapten coated cells	Anemia, cytopenia, thrombocytopenia	Variable

Type III (immune complex)	Tissue deposition of drug-antibody complexes with complement activation and inflammation	Serum sickness, vasculitis, fever, rash, arthralgia	1 to 3 weeks after drug exposure

Type IV (delayed, cell mediated)	MHC presentation of drug molecules to T cells with cytokine and inflammatory mediator release; may also be associated with activation and recruitment of eosinophils, monocytes, and neutrophils	Contact sensitivity Skin rashes, organ-tissue damage	2 to 7 days after drug exposure

IgE: immunoglobulin E; IgG: immunoglobulin G; IgM: immunoglobulin G; MHC: major histocompatibility complex

*These reactions may also be non-immunologically mediated.

Adapted from Riedl et al., 2003.^6^

Unlike immune-mediated drug reactions, pseudoallergic reactions are not associated with the production of antibodies or sensitized T cells, but are often clinically indistinguishable from drug hypersensitivity reactions. During these reactions, the drug has the ability, via its chemistry or pharmacology, to directly stimulate the release or activation of inflammatory mediators such as histamine (from mast cells, basophils), prostaglandins, leukotrienes, or kinins. Non-steroidal anti-inflammatory drugs (NSAIDs), narcotics, and angiotensin-converting enzyme (ACE) inhibitors are common causes of these non-allergic reactions [5,10,11].

## Risk factors

Factors associated with an increased risk of developing a drug allergy include age, gender, genetic polymorphisms, certain viral infections and drug-related factors (e.g., frequency of exposure, route of administration, molecular weight) (see Table 3). Drug allergy typically occurs in young and middle-aged adults, and is more common in women than men. Genetic polymorphisms in the human leukocyte antigen (HLA; a gene product of the major histocompatibility complex) as well as viral infections such as human immunodeficiency virus (HIV) and the Epstein-Barr virus (EBV), have also been linked to an increased risk of developing immunologic reactions to drugs. Susceptibility to drug allergy is influenced by genetic polymorphisms in drug metabolism. In addition, topical, intramuscular, and intravenous routes of administration are more likely to cause allergic drug reactions than oral administration; while intravenous administration is associated with more severe reactions. Prolonged high doses or frequent doses are more likely to lead to hypersensitivity reactions than a large single dose. Furthermore, large macromolecular drugs (e.g., insulin or horse antisera) or drugs that haptenate (bind to tissue or blood proteins and elicit an immune response), such as penicillin, are also associated with a greater likelihood of causing hypersensitivity reactions. Although atopic patients do not have an increased risk for drug allergy, they are at increased risk for serious allergic reactions [4,6,12-15].

**Table 3:** Risk factors for the development of drug allergy [15]

• **Patient-related factors:** • Age: young/middle-aged adults > infants/elderly • Gender: Women > men • Genetic polymorphisms • Viral infections: HIV, herpes viruses • Previous reaction to the drug
• **Drug-related factors:** • High molecular weight compounds and hapten-forming drugs are more immunogenic • Route: topical > intravenous/intramuscular > oral • Dose: frequent/prolonged > single dose

HIV: human immunodeficiency virus

## Diagnosis

The diagnosis of drug allergy requires a thorough history and the identification of physical findings and symptoms that are compatible with drug-induced allergic reactions. Depending on the history and physical examination results, diagnostic tests such as skin testing, graded challenges and induction of drug tolerance procedures may also be required. [1,4,6,15] Therefore, if drug allergy is suspected, evaluation by an allergist experienced in these diagnostic procedures is recommended.

### History

Evaluation of the patient with a suspected drug allergy should include a detailed history of all prescription and nonprescription drugs taken by the patient, including dates of administration, drug formulation, dosage and route of administration, clinical symptoms and their timing and duration in relation to drug exposure; as well as previous drug exposures and reactions [1,4,6,15].

### Clinical presentation

In addition to the detailed history, a careful physical examination can help to define possible mechanisms underlying the reaction and guide subsequent investigations and diagnostic testing. Table 4 highlights some of the most common clinical manifestations of drug allergy and examples of causative drugs.

**Table 4:** Clinical manifestations of drug allergy. [1,11,15]

Manifestation	Clinical Features	Examples of causative drugs
*Skin* Exanthemata	• Diffuse, fine macules and papules • Evolve over days post drug initiation	Allopurinol, penicillins, cephalosporins, anticonvulsants, sulfonamides

Urticaria, angioedema	• Onset within minutes to hours of drug administration • Potential for anaphylaxis • Often IgE-mediated	Antibiotics, ACE inhibitors, anticonvulsants, neuromuscular blocking agents, platinums, radiocontrast media, NSAIDs, narcotics

Fixed drug eruption	• Hyper-pigmented plaques that occur at the same site upon re-exposure to the culprit drug	Sulfonamide and tetracycline antibiotics, NSAIDs, ASA, sedatives, chemotherapeutic agents, anticonvulsants

SJS	• Fever, sore throat, fatigue, ocular involvement • Ulcers and other lesions on mucous membranes, particularly of the mouth and lips, as well as on truncal area	Sulfonamides, nevirapine, corticosteroids, anticonvulsants, NSAIDs (oxicams), allopurinol, phenytoin, carbamazepine, lamotrigine, barbiturates, psychotropic agents, pantoprazole, tramadol

TEN	• Similar to SJS, but usually involves significant epidermal detachment • Potentially life-threatening	Same as SJS

*Hematologic*	• Hemolytic anemia, leukopenia, thrombocytopenia	Penicillin, sulfonamides, anticonvulsants, cephalosporins, quinine, heparin, thiazides, gold salts

*Hepatic*	• Hepatitis, cholestatic jaundice	Sulfonamides, phenothiazines, carbamazepine, erythromycin, anti-tuberculosis agents, allopurinol, gold

*Renal*	• Interstitial nephritis, glomerulonephritis	Penicillin, sulfonamides, allopurinol, PPIs, ACE inhibitors, NSAIDs

*Multi-organ reactions* Anaphylaxis	• Urticaria/angioedema, bronchospasm, gastrointestinal symptoms, hypotension	Antibiotics, neuromuscular blocking agents, anesthetics, radiocontrast media, recombinant proteins (e.g., omalizumab)

DRESS	• Cutaneous eruption, fever, eosinophilia, hepatic dysfunction, lymphadenopathy	Anticonvulsants, sulfonamides, minocycline, allopurinol, strontium ranelate

Serum sickness	• Urticaria, arthralgias, fever	Heterologous antibodies, infliximab, allopurinol, thiazides, antibiotics (e.g., cefaclor) and bupropion

DILE	• Arthralgias, myalgias, fever, malaise	Hydralazine, procainamide, isoniazid, quinidine, minocycline, antibiotics, and anti–TNF-alpha agents

Vasculitis	• Cutaneous or visceral vasculitis	Sulfonamide antibiotics and diuretics, hydralazine, penicillamine, propylthiouracil

ACE: angiotensin-converting enzyme; NSAIDs: non-steroid anti-inflammatory drugs; SJS: Stevens-Johnson syndrome; TEN: toxic epidermal necrolysis; DRESS: Drug rash with eosinophilia and systemic symptoms; DILE: drug-induced lupus erythematosus; ASA: acetylsalicylic acid; PPIs: proton pump inhibitors; TNF: tumour necrosis factor

The skin is the organ most frequently and prominently affected by drug-induced allergic reactions [1,6,11]. The most common cutaneous manifestation is generalized exanthema (also known as a maculopapular rash), which is characterized by raised, spotted lesions that appears within days to 3 weeks after drug exposure, originate on the trunk, and eventually spread to the limbs. Urticaria (hives) and angioedema (swelling) are also common, and can results from both IgE-mediated and non-IgE-mediated mechanisms. The most severe forms of cutaneous drug reactions are Stevens-Johnson syndrome (SJS) and toxic epidermal necrolysis (TEN). SJS begins with a maculopapular rash that often progresses to bullae, mucous membrane ulcerations, conjunctivitis, fever, sore throat and fatigue. TEN is a rare condition with similar characteristics to SJS, but it also causes large portions of the epidermis (the skin’s outermost layer) to detach from the layers below, leading to extensive skin sloughing and a scalded skin appearance. Given the severity of these conditions, drugs suspected of causing SJS and TEN (most commonly sulfonamides) should be strictly avoided by the patient in the future [1].

Although skin reactions are the most common physical manifestation of drug-induced allergic reactions, many other organ systems may be involved, such as the renal, hepatic and hemolytic systems (see Table 4). Multi-organ reactions may also occur and include anaphylaxis (a serious systemic allergic reaction that is rapid in onset and may cause death; see Anaphylaxis article in this supplement), drug rash with eosinophilia and systemic symptoms (DRESS) syndrome, serum sickness, drug-induced lupus erythematosus (DILE) and vasculitis (a heterogeneous group of disorders that are characterized by inflammatory destruction of blood vessels). DRESS is a potentially life-threatening condition characterized by a widespread rash, fever, lymphadenopathy (swollen/enlarged lymph nodes) and hepatic dysfunction. Serum sickness is an immune-complex reaction that presents with fever, lymphadenopathy, arthralgia, and cutaneous lesions. The typical symptoms of DILE include sudden onset of fever and malaise; myalgia, arthralgia, and arthritis may also occur several weeks after drug initiation. In approximately 25% of cases, the skin may also be affected [1,11]. Serum sickness and DILE are usually self-limited, with symptoms resolving spontaneously within a few weeks after discontinuation of the offending drug. However, the symptoms of DRESS may worsen or persist for weeks, or even months, following drug discontinuation [1,11].

Since the clinical manifestations of drug allergy are highly variable, it is important to exclude other conditions that may mimic drug-induced allergic reactions. Table 5 lists some of the conditions that should be considered in the differential diagnosis of drug allergy.

**Table 5:** Conditions to consider in the differential diagnosis of drug allergy. [5]

**IgE-mediated drug allergy** **(urticaria, angioedema, anaphylaxis, bronchospasm):** • Carcinoid syndrome • Insect bites/stings • Mastocytosis • Asthma • Food allergy • Scombroid fish poisoning • Latex allergy • Infection (EBV, hepatitis A, B, C, gastrointestinal parasites)	**Non-IgE mediated reactions** **(exanthema, DRESS, SJS, TEN):** • Acute graft-versus-host disease • Kawasaki disease • Still’s disease • Psoriasis • Insect bites/stings • Viral infection • Streptococcal infection

IgE: immunoglobulin E; EBV: Epstein-Barr virus; SJS: Stevens-Johnson syndrome; TEN: toxic epidermal necrolysis; DRESS: Drug rash with eosinophilia and systemic symptoms

### Diagnostic tests

Skin testing procedures, such as skin prick testing (SPT) and intradermal tests (test in which the allergen is injected into the skin dermis) are useful for the diagnosis of IgE-mediated (type I) reactions. Skin testing protocols are standardized for penicillin, and are also useful (but rarely positive) for local anesthetics, muscle relaxants, and very sensitive for high-molecular-weight protein substances, such as insulin or monoclonal antibodies. Positive skin tests to these drugs confirm the presence of antigen-specific IgE and supports the diagnosis of a type I hypersensitivity reaction. The negative predictive value of penicillin skin testing is high with appropriate reagents and, therefore, a negative test result is useful for ruling out penicillin allergy. With other agents (except high molecular weight proteins), however, a negative skin test does not effectively rule out the presence of specific IgE. Serum-specific IgE tests are available for a limited number of drugs. However, these tests are costly and generally less sensitive than skin tests. Furthermore, most of these *in vitro* tests are not adequately validated for drug allergy testing [1,15].

Patch testing involves placing potential allergens (at non-irritant concentrations) on the patient’s back for 48 hours under aluminum discs, and then assessing for reactions. Drug patch testing is useful for the diagnosis of various delayed (type IV) cutaneous reactions, particularly exanthemata, but is generally not helpful for the diagnosis of SJS or TEN [1,10,11,15,16].

The measurement of histamine and tryptase levels have proved useful in confirming acute IgE-mediated reactions, particularly anaphylaxis; however, negative results do not rule out acute allergic reactions. A complete blood count can help diagnose hemolytic (type II) drug-induced reactions, such as hemolytic anemia, thrombocytopenia, or neutropenia. Hemolytic anemia may also be confirmed with a positive direct and/or indirect Coombs’ test (used to examine for the presence of antibodies on red blood cell membranes) [1,11,15].

Recent studies have focused on the potential role of the basophil activation test (the quantification of basophil activation by flow cytometry) in the diagnosis of drug allergy, since basophils are involved in both immune-mediated and non-immune-mediated reactions. Although some evidence suggests that the test is useful for evaluating possible allergies to beta-lactam antibiotics, NSAIDs and muscle relaxants, further confirmatory studies are needed before it is widely accepted as a diagnostic tool [1,17,18].

In cases where there is a definite medical need for a particular drug, but the clinical diagnosis of drug allergy remains uncertain despite thorough investigations, a procedure to induce temporary drug tolerance (also referred to as drug desensitization) or graded challenge testing (also known as provocation testing) may be considered. Induction of drug tolerance procedures temporarily modify a patient’s immunologic or non-immunologic response to a drug through the administration of incremental doses of the drug. Most regimens begin with a very dilute concentration of the drug, and the dose is doubled every 15 to 20 minutes, until a full therapeutic dose has been administered after 3 to 8 hours. Drug tolerance is usually maintained only as long as the drug is administered; the procedure needs to be repeated in the future if the patient requires the drug again after finishing a prior therapeutic course. Unlike induction of drug tolerance procedures, graded challenge tests do not modify a patient’s immunologic or non-immunologic response to a given drug. These tests are generally used to determine whether a patient will have an adverse reaction to a particular drug by administering sub-therapeutic doses over a period of time, while observing the patient for potential reactions. They are not advised if the patient has experienced a previous life-threatening reaction to the drug in question. Drug tolerance-induction procedures and graded challenges are potentially harmful and should only be performed by experienced personnel in facilities with resuscitative equipment readily available [1,19].

## Management of common drug allergies

The most effective strategy for the management of drug allergy is avoidance or discontinuation of the offending drug. When available, alternative medications with unrelated chemical structures should be substituted. Cross-reactivity among drugs should be taken into consideration when choosing alternative agents [1,11].

Additional therapy for drug hypersensitivity reactions is largely supportive and symptomatic. For example, topical corticosteroids and oral antihistamines may improve cutaneous symptoms. In the event of anaphylaxis, the treatment of choice is epinephrine administered by intramuscular injection into the lateral thigh. Systemic corticosteroids may also be used to treat severe systemic reactions, but should never be given prior to or replace epinephrine in the treatment of anaphylaxis. Severe drug reactions, such as SJS and TEN, are best treated in an intensive care or burn unit setting [1,11]. Strategies for the management of some of the most common drug allergies are discussed below.

### Penicillin

Penicillin is the most frequent drug allergy, affecting approximately 10% of patients. For patients with penicillin allergy, treatment is best limited to non-penicillin agents. Carbapenems (e.g., imipenem) do not exhibit a significant degree of cross-reactivity with penicillin and may be administered as a graded challenge after prophylactic skin tests with the relevant carbapenem [20,21]. Monobactams, such as aztreonam, are generally well tolerated by patients with penicillin allergy, except if they had an allergic reaction to ceftazidime [22-24]. Second- or third-generation cephalosporins may also be considered since the degree of cross reactivity with these agents and penicillin has been shown to be lower than with first-generation agents (see following Cephalosporin section) [1,25].

Ideally, management of the patient with penicillin allergy should include penicillin skin testing. Approximately 90% of patients have negative penicillin skin test responses and can safely receive cephalosporins as well as other beta-lactam agents. If a penicillin is deemed absolutely necessary in a penicillin-allergic patient, desensitization should be considered, and the procedure should only be performed under medical supervision in-hospital [1].

### Sulfonamides

Sulfonamide antibiotics are another common cause of drug-induced allergic reactions, and are often associated with delayed cutaneous maculopapular eruptions, SJS and TEN. Patients infected with HIV are at increased risk of developing cutaneous reactions to sulfonamide antibiotics, which is likely related to immunologic factors and frequent exposure to these antibiotics. Trimethoprim-sulfamethoxazole (TMP-SMX) is the drug of choice for the treatment of a number of HIV-associated infections and, therefore, many HIV-positive patients with a history of reacting to sulfonamides still require treatment with this antibiotic. Induction of drug tolerance procedures can be used to safely administer TMP-SMX to HIV-positive patients with a history of reacting to the antibiotic.

Since the chemical structure of non-antibiotic sulfonamides (e.g., thiazide diuretics, some NSAIDs and anticonvulsants) varies from sulfonamide antibiotics, these agents are not expected to cross-react, and can generally be safely administered to patients with a history of allergy to sulfonamide antibiotics. An exception is sulfasalazine which, by intestinal degradation becomes sulfapyridine, acquiring an aromatic immunogenic structure like sulfamethoxazole [1,26-28].

### Cephalosporins

The most common allergic reactions to cephalosporins are maculopapular rashes and drug fever; urticaria is less common and anaphylaxis is rare [25]. As mentioned earlier, positive skin tests to penicillin are associated with a higher likelihood of allergic reactions to first-generation cephalosporins. In these patients, alternate agents should be considered. In cephalosporin allergic subjects, there is limited cross reactivity on immunological testing between second- and third-generation cephalosporins and penicillins, especially amino-penicillins, but this has not necessarily indicated clinical reactivity [29]. There is a role for skin testing with the proposed antibiotic to be used in therapy, and/or administration by graded challenge. If skin testing is positive and no alternative drug exists, induction of drug tolerance procedures may be attempted [1,5].

### Radiocontrast media

Radiocontrast media (RCM) are associated with both allergic and pseudoallergic reactions. The incidence of reactions to RCM, including severe, life-threatening reactions, appears to be lower with non-ionic versus ionic agents. Pseudo/allergic reactions to RCM can usually be prevented through the use of pretreatment regimens that include oral corticosteroids and H1-antihistamines. Low osmolarity agents should also be used in such circumstances [1,5].

### Local anesthetics

True allergic reactions to local anesthetics (e.g., novocaine, lidocaine) are extremely rare; reactions are usually due to other ingredients in the medication, such as preservatives or epinephrine. However, if the reaction history is consistent with a possible immediate, IgE-mediated (type I) reaction, skin testing followed by graded challenge tests using epinephrine-free, preservative-free local anesthetics may be utilized [1].

### General anesthetics

Although rare, anaphylaxis may occur in patients under general anesthesia. The investigation of severe reactions during general anesthesia is particularly challenging given that the patient is often exposed to many co-administered drugs and agents. Reactions during general anesthesia are often due to neuromuscular blocking agents, but have also been associated with intravenous anesthetics (e.g., propofol, thiopentone, etomidate), antibiotics, NSAIDs and latex allergy. There are no reported cases of allergy to inhaled anesthetics. Assessment by an allergist is important for confirming the clinical diagnosis of allergy to general anesthesia, identifying likely causative agents as well as alternative agents that may be used safely in the future [30].

### Acetylsalicylic acid/NSAID reactions

Acetylsalicylic acid (ASA) and NSAIDs can cause both true allergic and pseudoallergic reactions, including exacerbations of underlying respiratory diseases, urticaria, angioedema, and anaphylaxis. Patients with underlying chronic respiratory diseases, such as asthma, rhinitis and sinusitis, may react to ASA and NSAIDs that inhibit cyclooxygenase-1 (COX-1). The management of these patients involves avoidance of aspirin and NSAIDs and aggressive treatment of the underlying respiratory disorder. Selective COX-2 inhibitors almost never cause reactions, and can typically be taken safely by patients with ASA/NSAID allergy. An induction of drug tolerance procedure to aspirin (also known as aspirin desensitization) may also be considered [1].

Patients with chronic urticaria/angioedema generally tolerate COX-2 inhibitors, but may experience exacerbations of urticaria/angioedema with NSAIDs that inhibit COX-1. True allergic reactions to NSAIDs are usually drug specific and, therefore, patients experiencing these reactions are often able to tolerate other NSAIDS [1].

## Prevention of future reactions

Prevention of future reactions is an essential part of patient management. The patient should be provided with written information about which drugs to avoid (including over-the-counter medications). The drugs should be highlighted in the hospital notes and within electronic records (where available), and the patient’s family physician should be informed of the drug allergy. Engraved allergy bracelets/necklaces, such as those provided by Medic Alert, should also be considered, particularly if the patient has a history of severe drug-induced allergic reactions [15].

## Conclusions

Drug allergy is a common clinical problem; assessment by an allergist is important for appropriate diagnosis and management of the condition. Diagnosis relies on a careful history and physical examination and, in some instances, skin testing, graded challenges and induction of drug tolerance procedures may be required. The mainstay of treatment for drug allergy is avoidance of the offending drug. When available, alternative medications with unrelated chemical structures should be substituted. Cross-reactivity among drugs should be taken into consideration when choosing alternative medications. If a particular drug to which the patient is allergic is indicated and there is no suitable alternative, induction of drug tolerance procedures may be considered to induce temporary tolerance to the drug.

## Key take-home messages

• Drug allergy encompasses a spectrum of immunologically mediated hypersensitivity reactions with varying mechanisms and clinical presentations.

• Risk factors for drug allergy include age (more common in young/middle-aged adults), gender (more common in females), genetic polymorphisms, certain viral infections (HIV and herpes viruses) and drug-related factors (topical and intravenous/intramuscular routes of administration are more immunogenic than oral administration).

• Referral to an allergist is important for appropriate diagnosis and treatment of drug allergy.

• Diagnosis requires a thorough drug history, including dates of administration, drug formulation, dosage and route of administration, as well as clinical symptoms and their timing and duration in relation to drug exposure; skin testing, graded challenges and induction of drug tolerance procedures may also be required.

• The skin is the organ most frequently affected by drug-induced allergic reactions, however, many other organ systems may be involved, including multi-organ reactions such as anaphylaxis.

• The mainstay of treatment is avoidance of the offending drug; alternative medications with unrelated chemical structures should be substituted when possible.

• If a particular drug to which the patient is allergic is indicated, induction of drug tolerance procedures may be considered to induce temporary tolerance to the drug.

## Competing interests

Dr. Richard Warrington is the past president of the Canadian Society of Allergy & Clinical Immunology and Editor-in-Chief of *Allergy*, *Asthma & Clinical Immunology.* He has received consulting fees and honoraria from Nycomed, CSL Behring and Talecris.

Dr. Fanny Silviu-Dan has no competing interests to disclose.
